# An efficient and secure attribute based signcryption scheme with LSSS access structure

**DOI:** 10.1186/s40064-016-2286-2

**Published:** 2016-05-17

**Authors:** Hanshu Hong, Zhixin Sun

**Affiliations:** Key Lab of Broadband Wireless Communication and Sensor Network Technology, Ministry Education, Nanjing University of Posts and Telecommunications, Nanjing, China

**Keywords:** Attribute based, Signcryption, LSSS structure, Security

## Abstract

Attribute based encryption (ABE) and attribute based signature (ABS) provide flexible access control with authentication for data sharing between users, but realizing both functions will bring about too much computation burden. In this paper, we combine the advantages of CP-ABE with ABS and propose a ciphertext policy attribute based signcryption scheme. In our scheme, only legal receivers can decrypt the ciphertext and verify the signature signed by data owner. Furthermore, we use linear secret sharing scheme instead of tree structure to avoid the frequent calls of recursive algorithm. By security and performance analysis, we prove that our scheme is secure as well as gains higher efficiency.

## Background

The notion of attribute based encryption (ABE) was first proposed by Sahai and Waters (2005). Since then, many typical ABE (Goyal et al. 2006; Waters 2011; Lewko et al. 2010; Goyal et al. 2008; Tian and Peng 2014) schemes have been proposed. In ABE, user’s access privileges are described by a set of attributes instead of a single identity string. A user can get access to the ciphertext only if his attributes satisfy with the policy which is set by the data owner. Due to its capability of providing fine-grained and flexible access control, ABE appears to be a promising tool for data encryption and data sharing between users. Attribute based signature (ABS) has been developed as a primitive to solve the data authentication problem of ABE, which was first introduced (Guo and Zeng 2008) in 2008. In ABS mechanisms (Maji et al. 2011), a signer can sign a message with the private key component corresponds with the attributes he processes. The signature can be verified to a certain set of attributes or an attribute access structure of which the data owner claims.

The notion of signcryption (Zheng 1997; Lim and Lee 1998; Tan 2008; Selvi et al. 2008) can be introduced to attribute based cryptography to present attribute based signcryption schemes. Signcryption (Paulo et al. 2005; Li and Khan 2012) is a single logical step to complete the function of both signature and encryption at the same time, thus it achieves better efficiency then the traditional sign-then-encryption method. However, research on attribute based signcryption has not been received much attention from academia. Wang and Huang (2011) proposed a signcryption scheme from pairings. Their scheme provides the same functions of encryption and authentication and is proved to be more efficient than the simply combination of “CP-ABE + CP-ABS”. Hu and Zhang (2013) proposed a fuzzy attribute based signcryption and apply it in the BAN (Body area network). Their scheme is a novel security mechanism and achieves outstand performance. However, the proposed (Wang and Huang 2011; Hu and Zhang 2013) schemes are based on the tree structure (Bethencourt et al. 2007) and threshold structure, which need frequent calls of recursive algorithm for the purpose of recovering the secret encryption component. Thus this will bring about external computation overhead.

To better improve the efficiency of attribute based signcryption scheme, in this paper, we propose an improved ciphertext policy attribute based signcryption scheme. We use LSSS structure (Beimel 1996) instead of access tree structure to avoid the frequent calls of recursive algorithm. By security and performance analysis, we prove that our scheme is secure as well as achieves higher efficiency.

## Preliminaries

### Bilinear pairings

Let $G_{1}$ and $G_{2}$ be two cyclic groups of prime order $q$. Let g be a generator of $G_{1}$. A bilinear pairing $\hat{e}$: $G_{1} \times G_{1} \to G_{2}$, $G_{2}$ has these features:

Bilinearity: for $a,b \in Z_{q}$, we have $\hat{e}\left( {g^{a} ,g^{b} } \right) = \hat{e}\left( {g,g} \right)^{ab}$.

Non-degeneracy: for any $g \in G_{1}$, $\hat{e}\left( {g,g} \right) \ne 1$.

Computability: the value of $\hat{e}\left( {u,v} \right)$ can be computed for any $u,v \in G_{1}$.

### Hardness assumption

#### Discrete logarithm assumption (DL)

Given $P,Q \in G_{1} ,$ no probabilistic polynomial-time (PPT) algorithm can find an integer $n \in Z_{q}^{*}$ such that $Q = P^{n}$ with non-negligible probability.

#### Decision bilinear Diffie–Hellman problem (DBDH)

For $a,b,c,z \in Z_{q}^{*}$, given $\{ g,g^{a} ,g^{b} ,g^{c} ,z\}$, no probabilistic polynomial-time (PPT) algorithm can distinguish the following tuples $\left\{ {A = g^{a} ,B = g^{b} ,C = g^{c} ,\hat{e}\left( {g,g} \right)^{abc} } \right\}$ and $\left\{ {A = g^{a} ,B = g^{b} ,C = g^{c} ,\hat{e}\left( {g,g} \right)^{z} } \right\}$ with non-negligible probability.

## Our model and assumptions

### Formulized definitions of our scheme

Our scheme consists of the following algorithms:

*Setup* On input security parameter, it returns the system public parameter $PK$ and master key $MK$. $PK$ is shared by users while $MK$ is kept private by the private key generator.

$Private\;Key\;generation$ On input the system public key $PK$, the master key $MK$, and an attribute set $\left\{ {A_{i} } \right\}$, private key generator (PKG) outputs $D_{i}$ as the user’s attribute private key. To distinguish the role of signers and receivers, in this paper, we define the private key of signer as $D_{s}$ while the private key of receiver as $D_{r}$.

$Signcrypt$ This algorithm is run by a signer which takes the systems public parameter $PK$, a plaintext $M$, signer’s private key $D_{s}$ and an access structure as input. Then it outputs the ciphertext $CT\left\{ {U,V,E} \right\}$.

$De {\text{-}} signcrypt$ This algorithm is run by the receiver. The algorithm takes as input the ciphertext $CT\left\{ {U,V,E} \right\}$ and the receiver’s private key $D_{r}$, it outputs either the plaintext $M$ or the reject symbol $\bot .$

### Security model

#### **Definition 1**

Our scheme has the essential confidentiality under chosen plaintext attack in selected model if no $Adversary$ has non-negligible advantage in the challenge game.

$Setup{\text :} \; Adversary$ claims a challenging attribute set $\gamma$. $Challenger$ runs setup algorithm to obtain $PK$. It sends $PK$ to $Adversary.$

$Adversary$ may make the following queries to $Challenger$.

$Private\;key\;generation\;query {\text :} \;Adversary$ can request the private key of an attribute set (expect for the challenging attribute set).

$Challenge{\text :} \;Adversary$ chooses two plaintexts $M_{0}$ and $M_{1}$. $Challenger$ chooses $\mu \in \left\{ {0,1} \right\}$ randomly and calculates $C^{*} = Signcrypt\left\{ {PK,M_{\mu } ,D_{s} } \right\}$. Then $Challenger$ sends the result back to $Adversary$.

$Adversary$ cannot ask $Challenger$ for $Private\;key\;generation$ query for the challenging attribute set $\gamma$.

$Adversary$ outputs a value $\mu^{*}$ as a conjecture of $\mu$. If $\mu^{*} = \mu$ then $Adversary$ wins the game.

Denote $\left| {\Pr \left[ {\mu^{*} = \mu } \right] - \frac{1}{2}} \right|$ to be the advantage of $Adversary$.

#### Definition 2

Our scheme has the existential unforgeability under chosen message attack in the selective model if no $Adversary$ has non-negligible advantage in the challenge game.

$Setup {\text :} \;Adversary$ claims a challenging attribute set $\gamma$. $Challenger$ takes a security parameter and runs setup procedure to obtain the system parameters. It sends the $PK$ to $Adversary$.

$Private\;key\;generation\;query {\text :} \;Adversary$ can request the private key of an attribute set (expect for the challenging attribute set).

$Signcrypt query{\text :} \;Adversary$ chooses an attribute set $\left\{ {A_{i} } \right\}$, an access structure, a plaintext M. $Challenger$ calculates $D_{s}$ and runs the signcrypt procedure to calculate the ciphertext $CT = Signcrypt\left\{ {PK,M,D_{i} ,\gamma } \right\}$. After then, $Challenger$ sends $CT$ to $Adversary$.

$$Challenge$$: $$Adversary$$ computes a 3-tuple $$CT^{*} \left\{ {U,V,E} \right\},$$ while $$CT^{*} \left\{ {U,V,E} \right\}$$ was not from a $$igncrypt$$$$query$$.

$Challenger$ de-signcrypts the ciphertext by running the $De{ {\text{-}} }signcrypt\,\{ PK,CT^{*} ,D_{r}$ }.

$Adversary$ wins the game if the output of $De{ {\text{-}} }signcrypt$ is not $\bot$.

Denote $Adv\left( A \right) = \left| {\Pr \left[ {Result = M} \right]} \right|$ to be the advantage of $Adversary$.

## Our contributions to attribute based signcryption scheme

Let $G_{1}$ and $G_{2}$ be two cyclic groups of prime order $p$, while *g* is the generator of $G_{1}$. Let $\hat{e} :G_{1} \times G_{1} \to G_{2}$ be a bilinear pairing. Define 2 functions: $H_{1} , H_{2} .$ The function $H_{1}$ associates attributes to rows of access $Matrix$ (the number of rows $\in Z_{p}^{*}$). $H_{2} :\left\{ {0,1} \right\}^{n} \to Z_{p}^{*}$.

$Setup$ PKG randomly chooses $\alpha_{i} \in Z_{p}^{*}$ for each attribute $i$ in the system. Besides, PKG chooses another secret number $\alpha \in Z_{p}^{*} .$ The system outputs the system master keys $\left\{ {g^{\alpha } ,\alpha_{i} } \right\}$, public parameters $\left\{ {\hat{e}\left( {g,g} \right)^{\alpha } ,\hat{e}\left( {g,g} \right)^{{\alpha_{i} H_{1} \left( i \right)}} ,H_{1} ,H_{2} ,G_{1} ,G_{2} ,p,g} \right\}$.

$Private\;key\;generation$ For signer’s attribute set $\left\{ {A_{j} } \right\}$, PKG chooses $u \in Z_{p}^{*}$ and calculates its private key $\left\{ {D_{s,1} ,D_{s,2} ,D_{s,3} } \right\} = \left\{ {g^{{u + \alpha_{j} H_{1} \left( j \right)}} ,g^{\alpha + u} ,\hat{e}\left( {g,g} \right)^{u} } \right\}$. Likewisely, for receiver’s attribute set $\left\{ {A_{i} } \right\}$ PKG chooses $h \in Z_{p}^{*}$ calculates its private key $\left\{ {D_{r,1} ,D_{r,2} ,D_{r,3} } \right\} = \left\{ {g^{{\alpha_{i} H_{1} \left( i \right) + h}} ,g^{\alpha + h} ,\hat{e}\left( {g,g} \right)^{h} } \right\}.$ PKG transfers the private key to each user through secure channels.

$Signcrypt$ Signer firstly picks $x \in Z_{p}^{*}$ and a LSSS access structure $Matrix,$ then chooses random vector $\vec{v} = \left( {x,vr_{1} ,vr_{2} , \ldots ,vr_{n} } \right) \in Z_{p}^{n} .$ Let $\lambda_{i} = \vec{v} \cdot Matrix_{i}$. ($Matrix_{i}$ stands for the $i{\text{th}}$ row of the corresponding $Matrix$). Finally, singer randomly picks $r_{i} \in Z_{p}^{*}$ and calculates the signcryption information:

$$U = \left\{ {\hat{e}\left( {g,g} \right)^{{\mathop \sum \nolimits_{j \in S} \alpha_{j} H_{1} \left( j \right) \cdot x}} } \right\}$$

$$t = H_{2} (U||M)$$

$$V:\left\{ {v_{1} = \mathop \prod \limits_{j \in S} D_{s,1}^{x + t} , v_{2} = \mathop \prod \limits_{j \in S} D_{s,3}^{x + t} } \right\}$$

1 $$E:\left\{ {C_{0} = M\hat{e}\left( {g,g} \right)^{\alpha x} ,C_{1} = g^{x} ,C_{2,i} = \hat{e}\left( {g,g} \right)^{{ - \alpha_{i} H_{1} \left( j \right) \cdot \lambda_{i} }} ,C_{3,i} = g^{{\lambda_{i} }} } \right\}$$

Signer sends $CT = \left\{ {U,V,E} \right\}$ to the receiver.

$De{\text{-}}signcrypt$ Let $\left\{ {\omega \in Z_{p} } \right\}_{i \in l}$ be a set of constants such that if $\{ \lambda_{i} \}$ are valid shares of secret $x$ according to $Matrix$, then $\mathop \sum \nolimits_{i \in l} \omega_{i} \lambda_{i} = x$. Receiver calculates $M^{*}$ as follows:

2 $$M^{*} = \frac{{C_{0} }}{{\mathop \prod \nolimits_{i \in l} \left( {\hat{e}\left( {C_{3} } \right.,D_{r,1} ) \cdot C_{2,i} } \right)^{{\omega_{i} }} \cdot \hat{e}\left( {C_{1} } \right.,D_{r,2} )}}$$

Then, receiver verifies if

3 $$\hat{e}\left( {v_{1} ,g } \right) = U \cdot v_{2} \cdot \hat{e}\left( {g,g} \right)^{{\mathop \sum \nolimits_{j \in S} \alpha_{j} H_{1} \left( j \right) \cdot t}}$$

If Eq. (3) holds then the algorithm outputs plaintext $M$ with the signature. If not, it outputs reject “$\bot$”.

Correctness proof:

(a) Decryption:

4 $$\begin{aligned} M^{*} &= \frac{{C_{0} }}{{\mathop \prod \nolimits_{i \in l} \left( {\hat{e}\left( {C_{3,i} } \right.,D_{r,1} ) \cdot C_{2,i} } \right)^{{\omega_{i} }} \cdot \hat{e}\left( {C_{1} } \right.,D_{r,2} )}} = \frac{{C_{0} \cdot \hat{e}\left( {C_{1} } \right.,D_{r,2} )^{ - 1} }}{{\mathop \prod \nolimits_{i \in l} \left( {\hat{e}\left( {g^{{\lambda_{i} }} ,g^{{\alpha_{i} H_{1} \left( i \right) + u}} } \right)\hat{e}\left( {g,g} \right)^{{ - \alpha_{i} H_{1} \left( j \right) \cdot \lambda_{i} }} } \right)^{{\omega_{i} }} }} \\ &= \frac{{C_{0} \cdot \hat{e}\left( {C_{1} } \right.,D_{r,2} )^{ - 1} }}{{\mathop \prod \nolimits_{i \in l} \left( {\hat{e}\left( {g,g} \right)^{{u\lambda_{i} }} } \right)^{{\omega_{i} }} }} \\ &= \frac{{M\hat{e}\left( {g,g} \right)^{\alpha x} \cdot \hat{e}\left( {g,g} \right)^{ux} }}{{\hat{e}\left( {g,g} \right)^{\alpha x} \cdot \hat{e}\left( {g,g} \right)^{{u\mathop \sum \nolimits_{i \in l} \lambda_{i} \omega_{i} }} }} \\ &= M \\ \end{aligned}$$

(b) Signature verification:

5 $$\begin{aligned} t &= H_{2} (U||M) \\ \hat{e}\left( {v_{1} ,g } \right) &= \hat{e}\left( {g^{{\mathop \sum \nolimits_{j \in S} \left( {\alpha_{j} H_{1} \left( j \right) + u} \right) \cdot \left( {x + t} \right)}} ,g} \right) \\ &= \hat{e}\left( {g,g} \right)^{{\mathop \sum \nolimits_{j \in S} \alpha_{j} H_{1} \left( j \right) \cdot \left( {x + t} \right)}} \cdot \hat{e}\left( {g,g} \right)^{{\mathop \sum \nolimits_{j \in S} u\left( {x + t} \right)}} \\ &= \hat{e}\left( {g,g} \right)^{{\mathop \sum \nolimits_{j \in S} \alpha_{j} H_{1} \left( j \right) \cdot x}} \cdot \hat{e}\left( {g,g} \right)^{{\mathop \sum \nolimits_{j \in S} \alpha_{j} H_{1} \left( j \right) \cdot t}} \cdot \hat{e}\left( {g,g} \right)^{{\mathop \sum \nolimits_{j \in S} u\left( {x + t} \right)}} \\ &= U \cdot v_{2} \cdot \hat{e}\left( {g,g} \right)^{{\mathop \sum \nolimits_{j \in S} \alpha_{j} H_{1} \left( j \right) \cdot t}} \\ \end{aligned}$$

## Security and efficiency analysis

### Confidentiality

**Theorem 1*** If*$Adversary$* can break our scheme under chosen plaintext attack in the selective model, then a simulator can solve the DBDH problem*.

*Proof* In the challenge game, if there exists an $Adversary$ which has advantage $\varepsilon$ in attacking our scheme, there exists a simulator solving the DBDH problem with an advantage of $\varepsilon/2$.

The simulator is constructed as follows:

Phase 1 $Setup {\text :} \;Adversary$ claims a challenging attribute set $\gamma$. $Challenger$ defines a set of attributes $\left\{ {A_{i} } \right\}.$ Let $G_{1}$ and $G_{2}$ be two cyclic groups of prime order $p$,while *g* is the generator of $G_{1}$. Let $\hat{e} :G_{1} \times G_{1} \to G_{2}$ be a bilinear pairing. Define 2 functions $: H_{1}$ associates attributes to rows of access $Matrix$, $H_{2} :\left\{ {0,1} \right\}^{*} \to Z_{p}^{*} .$

$$Challenger$$ randomly chooses $$\mu \in \left\{ {0,1} \right\}$$, $$a,b,c \in Z_{p}^{*}$$.

$${\text{Let}}\;\left\{ {\begin{array}{*{20}l} {\left( {A,B,C,Z} \right) = \left( {g^{a} ,g^{b} ,g^{c} ,\hat{e}\left( {g,g} \right)^{abc} } \right)\quad if \mu = 0} \hfill \\ {\left( {A,B,C,Z} \right) = \left( {g^{a} ,g^{b} ,g^{c} ,\hat{e}\left( {g,g} \right)^{z} } \right)\quad if \mu = 1} \hfill \\ \end{array} } \right.$$

The aim of simulator is to output a value $\mu^{*}$ as a conjecture of $\mu .$

The simulator simulates the role of $Challenger$ and runs $Adversary$’s algorithm as subprogram.

Phase 2 $$Queries$$:

$Adversary$ asks for private key for attributes $A_{i}$. Simulator picks $u,y,a_{i} \in Z_{p}^{*}$ and makes the following settings:

6 $$\left\{ {D_{r,1} ,D_{r,2} ,D_{r,3} } \right\} = \left\{ {\begin{array}{*{20}c} {g^{{u + \alpha_{i} H_{1} \left( i \right)}} ,g^{ab + u} ,\hat{e}\left( {g,g} \right)^{u} ,\quad\; if A_{i} \in \gamma } \\ {g^{{u + \alpha_{i} H_{1} \left( i \right)}} ,g^{y + u} ,\hat{e}\left( {g,g} \right)^{u} ,\quad if \;A_{i} \notin \gamma } \\ \end{array} } \right.$$

The queries like Phase 2 can be asked by $Adversary$ for a bounded times.

Phase 3 $$Challenge$$:

$Adversary$ picks plaintext $M_{0}$, $M_{1}$ and a challenging LSSS containing attribute set $\gamma$.

Simulator chooses $\mu \in \left\{ {0,1} \right\}$ and calculates $CT_{\mu } = Signcrypt\left\{ {PK,M_{\mu } ,D_{s} } \right\}$.

Simulator sends $CT_{\sigma }$ to $Adversary$.

$$CT_{\mu } :\left\{ {C_{0} = M\hat{e}\left( {g,g} \right)^{abx} ,C_{1} = g^{x} ,C_{2,i} = \hat{e}\left( {g,g} \right)^{{ - \alpha_{i} H_{1} \left( j \right) \cdot \lambda_{i} }} ,C_{3,i} = g^{{\lambda_{i} }} } \right\}$$

Let $x = c$, accoding to the previous setting in the $Setup$ phase:

7 $$CT_{\mu } = \left\{ {\begin{array}{*{20}l} {M\hat{e} \left( {g,g} \right)^{abc} ,\quad if \mu = 0} \hfill \\ {M\hat{e} \left( {g,g} \right)^{z} ,\quad if \mu = 1} \hfill \\ \end{array} } \right.$$

$$Adversary$$ outputs a value $$\mu^{*}$$ as a guess of $$\mu .$$ If $$\mu^{*} = \mu$$$$Adversary$$ wins the game.

Then we will discuss simulator’s advantage in distinguishing the following two tuples $\left\{ {A = g^{a} ,B = g^{b} ,C = g^{c} ,\hat{e}\left( {g,g} \right)^{abc} } \right\}$ and $\left\{ {A = g^{a} ,B = g^{b} ,C = g^{c} ,\hat{e}\left( {g,g} \right)^{z} } \right\}$.

When $\mu = 1$, $E$ is a illegal ciphertext and $Adversary$ cannot acquire useful information of $\sigma .$

8 $$Pr\left( {\mu^{*} \ne \mu |\mu = 1} \right) = \frac{1}{2}$$

Since when $\mu^{*} \ne \mu$, the simulator outputs $\mu = 1$, so:

9 $$Pr\left( {\mu^{*} = \mu |\mu = 1} \right) = \frac{1}{2}$$

When $\mu = 0$, $E$ is a legal ciphertext. According to the assumption, $Adversary$ has an advantage $\varepsilon .$

10 $$Pr\left( {\mu^{*} = \mu |\mu = 1} \right) = \frac{1}{2} + \varepsilon$$

Since when $\mu^{*} = \mu$ the simulator outputs $\mu = 1$, so

11 $$Pr\left( {\mu^{*} = \mu |\mu = 0} \right) = \frac{1}{2} + \varepsilon$$

As is mentioned above, the advantage of simulator is

12 $$\begin{aligned} & \frac{1}{2}Pr\left( {\mu^{*} = \mu |\mu = 0} \right) + \frac{1}{2}Pr\left( {\mu^{*} = \mu |\mu = 1} \right) - \frac{1}{2} \\ & \quad = \frac{1}{2}\left( {\frac{1}{2} + \varepsilon } \right) + \frac{1}{2} \times \frac{1}{2} - \frac{1}{2} \\ & \quad = \frac{\varepsilon }{2} \\ \end{aligned}$$

### Unforgeability

**Theorem 2*** If an*$Adversary$* can break our scheme chosen message attack in the selective model, then it can be constructed that a simulator with a non- negligible advantage solves the DBDH problem*.

*Proof* In the challenge game, if there exists an $Adversary$ which has advantage $\varepsilon$ in forging a legal ciphertext, there exists a simulator which can solve the DBDH problem with an advantage of $\varepsilon/2$.

Phase 1 $$Setup {\text :}$$

$Adversary$ claims a challenging attribute set $\gamma$. $Challenger$ defines a set of attributes $\left\{ {A_{i} } \right\}$; Let $G_{1}$ and $G_{2}$ be two cyclic groups of prime order $p$, while *g* is the generator of $G_{1}$. Let $\hat{e}:G_{1} \times G_{1} \to G_{2}$ be a bilinear pairing. Define 2 functions: $H_{1}$ associates attributes to rows of access $Matrix,H_{2} :\left\{ {0,1} \right\}^{*} \to Z_{p}^{*} .$

$$Challenger$$ randomly chooses $$b \in \left\{ {0,1} \right\}$$, $$a,b,c \in Z_{p}^{*}$$.

$${\text{Let}}\;\left\{ {\begin{array}{*{20}c} {\left( {A,B,C,Z} \right) = \left( {g^{a} ,g^{b} ,g^{c} ,\hat{e}\left( {g,g} \right)^{abc} } \right)\quad if \mu = 0} \\ {\left( {A,B,C,Z} \right) = \left( {g^{a} ,g^{b} ,g^{c} ,\hat{e}\left( {g,g} \right)^{z} } \right)\quad if \mu = 1} \\ \end{array} } \right.$$

The aim of simulator is to output a value $\mu^{*}$ as a conjecture of $\mu$.

Phase 2 $$Queries{\text :}$$

$Private\;key\;generation\;query {\text :} \;Adversary$ chooses a set of attributes $\left\{ {A_{j } } \right\}$, a plaintext $M$ and a LSSS. Simulator picks $u,y,a_{i} ,b_{i} ,y_{i} \in Z_{p}^{*}$ and makes the following settings:

13 $$\left\{ {D_{s,1} ,D_{s,2} ,D_{s,3} } \right\} = \left\{ {\begin{array}{*{20}l} {g^{{u + \alpha_{i} b_{i} H_{1} \left( i \right)}} ,g^{ab + u} ,\hat{e}\left( {g,g} \right)^{u} ,\quad if \;A_{j} \in \gamma } \hfill \\ {g^{{u + y_{i} H_{1} \left( i \right)}} ,g^{y + u} ,\hat{e}\left( {g,g} \right)^{u} ,\quad if\; A_{j} \notin \gamma } \hfill \\ \end{array} } \right.$$

$$Signcrypt$$$$query$$: $$Adversary$$ picks a message $$M$$ for signcrypt query. Simulator runs algorithm $$Signcrypt \left\{ {M,D_{s} ,PK} \right\}$$ and returns the result $$CT = \{ U,V,E\}$$ to $$Adversary.$$

The queries like Phase 2 can be asked by $Adversary$ for a bounded times.

Phase 3 $$C{{h}}allenge{\text :}$$

$Adversary$ outputs a ciphertext $CT^{*} \{ U^{*} ,V^{*} ,E^{*} \}$. $Adversary$ makes the forges the illegal ciphertext as the following process:

$$U^{*} = \left\{ {\begin{array}{*{20}l} {\hat{e}\left( {g,g} \right)^{{a_{i} b_{i} H_{1} \left( j \right) \cdot x}} ,\quad A_{j} \in \gamma } \hfill \\ {\hat{e}\left( {g,g} \right)^{{y_{i} H_{1} \left( i \right) \cdot x}} ,\quad A_{j} \notin \gamma } \hfill \\ \end{array} } \right.$$

$$t = H_{2} \left( {U^{*} ||M} \right)$$

$$V = \left\{ {v_{1}^{*} ,v_{2}^{*} } \right\} = \left\{ {\begin{array}{*{20}l} {g^{{\left( {\alpha_{j} b_{j} H_{1} \left( j \right) + u} \right)^{*} \cdot \left( {x + t} \right)}} ,\hat{e}\left( {g,g} \right)^{{u \cdot \left( {x + t} \right)}} ,\quad A_{j} \in \gamma } \hfill \\ {g^{{\left( {y_{j} H_{1} \left( j \right) + u} \right)^{*} \cdot \left( {x + t} \right)}} ,\hat{e}\left( {g,g} \right)^{{u \cdot \left( {x + t} \right)}} ,\quad A_{j} \notin \gamma } \hfill \\ \end{array} } \right.$$

14 $$E^{*} {\text :} \left\{ {C_{0} = M\hat{e}\left( {g,g} \right)^{abx} ,C_{1} = g^{x} ,C_{2,i} = \hat{e}\left( {g,g} \right)^{{ - \alpha_{i} H_{1} \left( j \right) \cdot \lambda_{i} }} ,C_{3,i} = g^{{\lambda_{i} }} } \right\}$$

Simulator verifies the ciphertext $CT^{*} \left\{ {U^{*} ,V^{*} ,E^{*} } \right\}$. Simulator firstly calculates the legal private key of receivers’ attribute set $\left\{ {A_{i} } \right\}$:

15 $$\{ D_{s,1} ,D_{s,2} \} = \left\{ {\begin{array}{*{20}l} {\left\{ {g^{{a_{j} b_{j} H_{1} \left( j \right) + u}} ,g^{ab + u} ,A_{j} \in \gamma } \right\}} \hfill \\ {\left\{ {g^{{y_{j} H_{1} \left( j \right) + u}} ,g^{y + u} ,A_{j} \notin \gamma } \right\}} \hfill \\ \end{array} } \right.$$

Then decrypts and verifies:

$$M^{*} = \frac{{C_{0} }}{{\mathop \prod \nolimits_{i \in l} \left( {\hat{e}\left( {C_{3} } \right.,D_{r,1} ) \cdot C_{2,i} } \right)^{{\omega_{i} }} \cdot \hat{e}\left( {C_{1} } \right.,D_{r,2} )}},$$

$$t = H_{2} \left( {U||M} \right)$$

16 $$\begin{aligned} \hat{e}\left( {v_{1}^{*} ,g } \right) &= \left\{ {\begin{array}{*{20}c} {\hat{e}\left( {g^{{(a_{j} b_{j} H_{1} \left( j \right) + u)\left( {x + t} \right)}} ,g} \right),\quad A_{j} \in \gamma } \\ {\hat{e}\left( {g^{{(y_{j} H_{1} \left( j \right) + u)\left( {x + t} \right)}} ,g} \right),\quad A_{j} \notin \gamma } \\ \end{array} } \right. \\ &= \left\{ {\begin{array}{*{20}l} {\hat{e}\left( {g,g} \right)^{{\alpha_{j} b_{j} H_{1} \left( j \right) \cdot x}} \cdot \hat{e}\left( {g,g} \right)^{{\alpha_{j} b_{j} H_{1} \left( j \right) \cdot t}} \cdot \hat{e}\left( {g,g} \right)^{{u\left( {x + t} \right)}} ,\quad A_{j} \in \gamma } \hfill \\ {\hat{e}\left( {g,g} \right)^{{y_{j} H_{1} \left( j \right) \cdot x}} \cdot \hat{e}\left( {g,g} \right)^{{y_{j} H_{1} \left( j \right) \cdot t}} \cdot \hat{e}\left( {g,g} \right)^{{u\left( {x + t} \right)}} ,\quad A_{j} \notin \gamma } \hfill \\ \end{array} } \right. \\ \end{aligned}$$

Let $f = \hat{e}\left( {g,g} \right)^{{\alpha_{j} b_{j} H_{1} \left( j \right) \cdot t + u\left( {x + t} \right)}} ,g^{{H_{1} \left( j \right) \cdot x}} = g^{c}$, according to the previous setting in the $Setup$ phase:

17 $$\hat{e}\left( {v_{1}^{*} ,g } \right) = \left\{ {\begin{array}{*{20}l} {f \cdot v_{2}^{*} \cdot \hat{e}\left( {g,g} \right)^{abc} ,\quad if \;u = 0} \hfill \\ {f \cdot v_{2}^{*} \cdot \hat{e}\left( {g,g} \right)^{z} , \quad if\; u = 1 } \hfill \\ \end{array} } \right.$$

When $\mu = 1$, $\hat{e}\left( {v_{1}^{*} ,g } \right)$ is a random number and $Adversary$ fails to forge a legal ciphertext.

18 $$Pr\left( {\mu^{*} = \mu |\mu = 1} \right) = \frac{1}{2}$$

When $\mu = 0$, $E$ is a legal ciphertext and $Adversary$ successfully forges the ciphertext. According to the assumption, $Adversary$ has an advantage $\varepsilon .$

19 $$Pr\left( {\mu^{*} = \mu |\mu = 0} \right) = \frac{1}{2} + \varepsilon$$

As is mentioned above, the advantage of simulator is

20 $$\begin{aligned} & \frac{1}{2}Pr\left( {\mu^{*} = \mu |\mu = 0} \right) + \frac{1}{2}Pr\left( {\mu^{*} = \mu |\mu = 1} \right) - \frac{1}{2} \\ & \quad = \frac{1}{2}\left( {\frac{1}{2} + \varepsilon } \right) + \frac{1}{2} \times \frac{1}{2} - \frac{1}{2} \\ & \quad = \frac{\varepsilon }{2} \\ \end{aligned}$$

### Efficiency analysis

In this paper, we compare the proposed scheme with Wang’s and Hu’s schemes with respect to the computation cost and access control method. Due to the fact that the computation cost of add operation and multiply operation is much smaller than that of exponential operation and bilinear pairing operation, consequently, we mainly compare the number of exponential operation and bilinear pairing operation in different schemes. We denote “Exp” and “Pair” by exponential operation and bilinear pairings. Detailed results are listed in Table 1.

**Table 1:** Performance comparison

Schemes	Access control method	Signcryption computation cost	De-signcryption computation cost
Wang and Huang (2011)	Access tree	2 Exp + 1Pair	(1 + 2nlog *n*)Exp + (4n + 1)Pair
Hu and Zhang (2013)	Threshold	(2n + 5) Exp	2n Exp + (3n + 2)Pair
Our scheme	LSSS matrix	(5n + 2) Exp	(n + 1) Exp + (2n + 1)Pair

From Table 1, we can figure out that the number of exponential operation in the signcryption in our CP-ABSC is more than those in Wang and Huang (2011) and Hu and Zhang (2013), however, the number of bilinear pairing operation in the de-signcryption is decreased greatly. Since the computation burden of bilinear pairing operation is heavier than that of exponential operation, the total computation cost has been reduced in our scheme. What’s more, our CP-ABSC adopts LSSS to realize data access control, which differs from the access structures in Wang and Huang (2011 and Hu and Zhang (2013). The LSSS access structure not only avoids the frequent calls of recursive algorithm used in access tree structure model, but also provides more flexible control management and increases the overall efficiency of the cryptosystem.

## Conclusion

In this paper, we propose an optimized attribute based signcryption scheme. By security analysis, we prove that it meets the security demands of confidentiality, unforgeability and non-repudiation. Besides, by introducing LSSS structure to implement the access control function, the flexibility and efficiency of the whole attributed based signcryption system has been improved.

Our future work should focus on the attribute revocation and key refreshing in the attribute based encryption. Since users with the same set of attributes share the same private key, once a single user’s private key has been leaked, a group of users’ privacy and privilege will be damaged. Consequently, protecting users’ privacy and refreshing private keys at a lower cost when private key leakage happens is a problem urgently to be solved and should be taken into our future research direction.
